# Supplementary material for: Deciphering High-Temperature-Induced Lignin Biosynthesis in Wheat through Comprehensive Transcriptome Analysis
Source: Plants (Basel). 2024 Jul 3;13(13):1832. doi: 10.3390/plants13131832 (PMC11243994; doi:10.3390/plants13131832)
Supplement: Supplementary file 1 [file plants-13-01832-s001.zip › Supplementary Figures Several figures cited in the article.pdf]

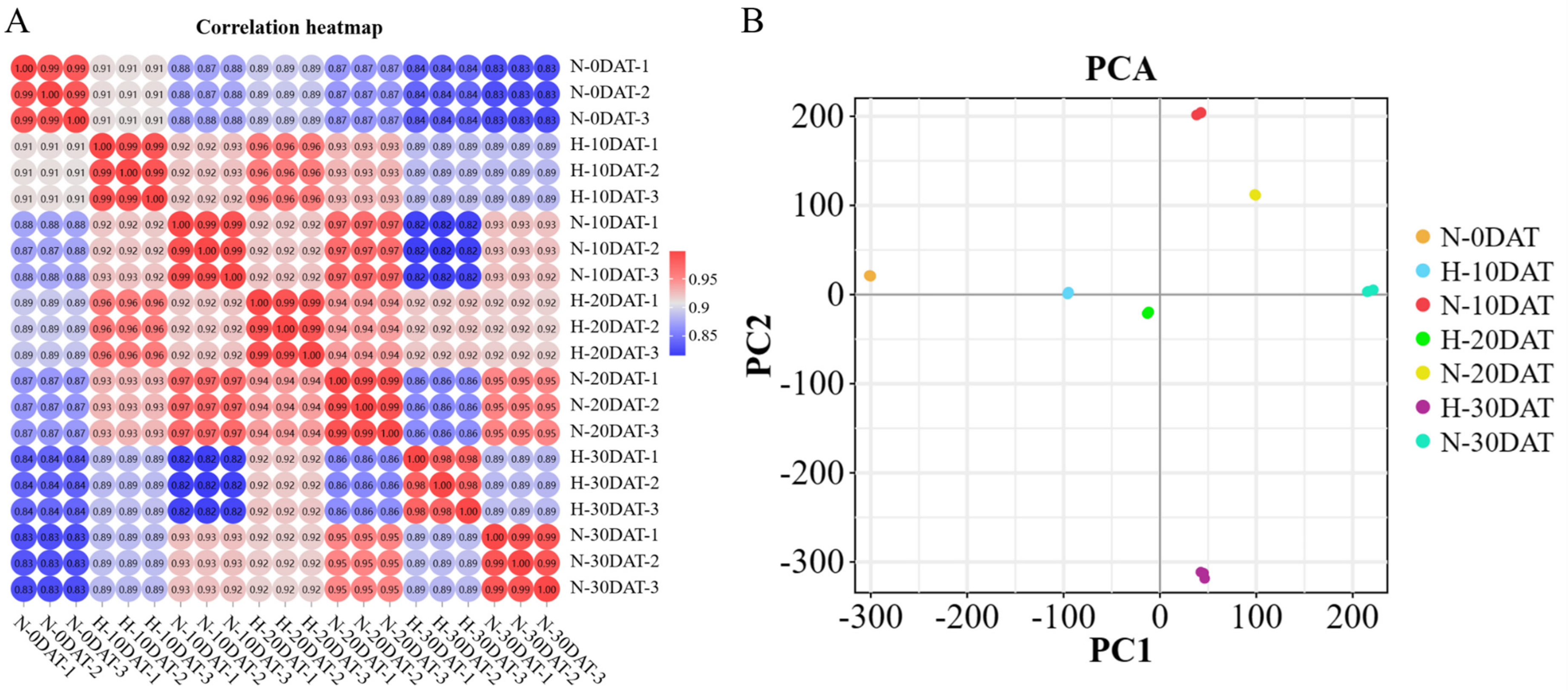

Supplementary Figure S1 Correlation and PCA analysis of all transcripts. A, Utilize the Pearson correlation coefficient to assess the correlation among 21 samples, where the intensity of the color represents the strength of the correlation. A deeper red signifies a stronger correlation, while a lighter color indicates a weaker correlation. B, PCA plot of transcriptome results.

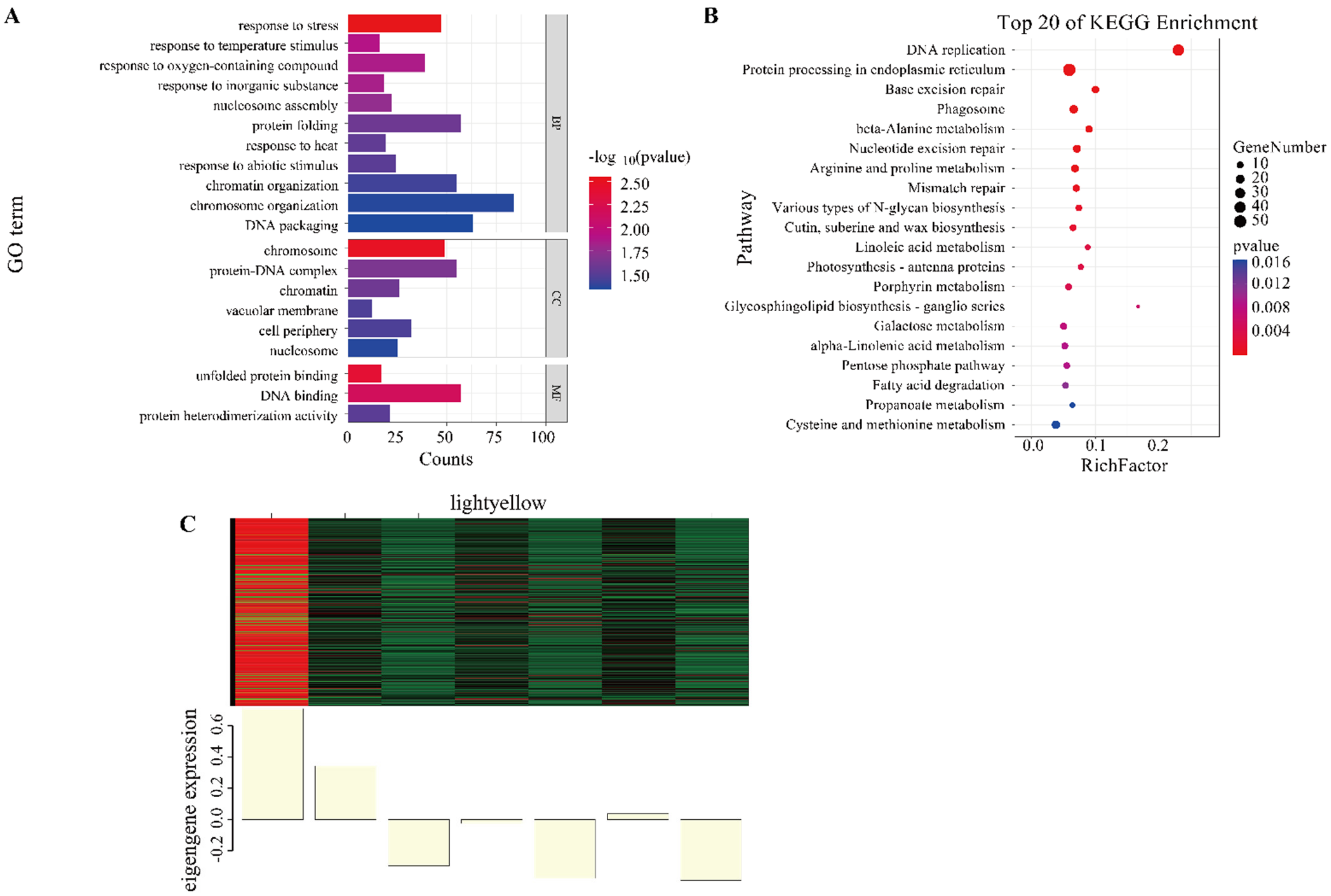

Supplementary Figure S2 MElightyellow-related genes and their annotations. **A,** Significant GO terms in MElightyellow. **B,** Significant KEGG terms in MElightyellow. **C**, Eigengene expression during each treatment period in MElightyellow; heatmap from green to red for expression from high to low, respectively.

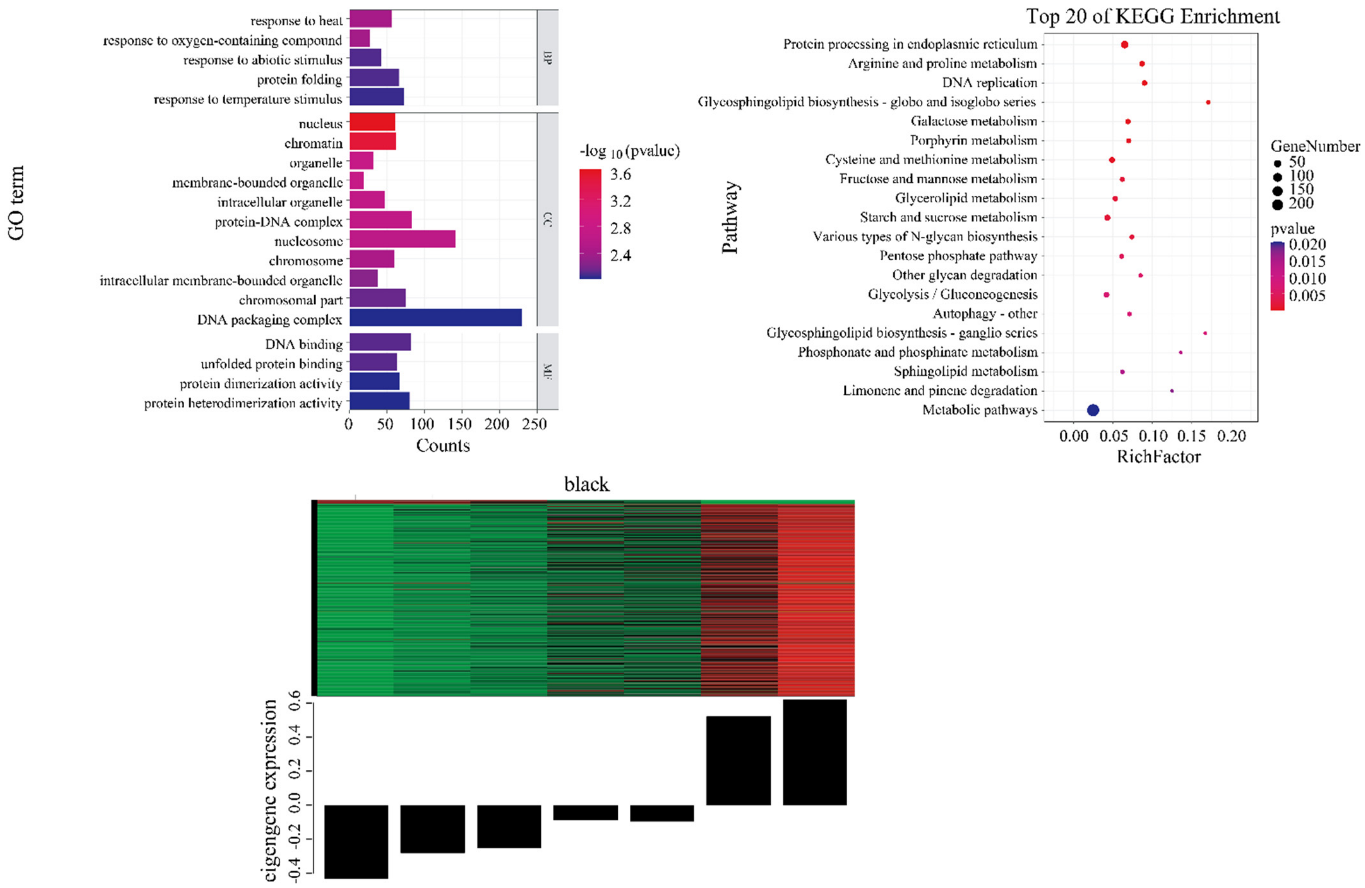

Supplementary Figure S3 MEblack-related genes and their annotations. **A,** Significant GO terms in MEblack. **B,** Significant KEGG terms in MEblack. **C**, Eigengene expression during each treatment period in MEblack; heatmap from green to red for expression from high to low, respectively.

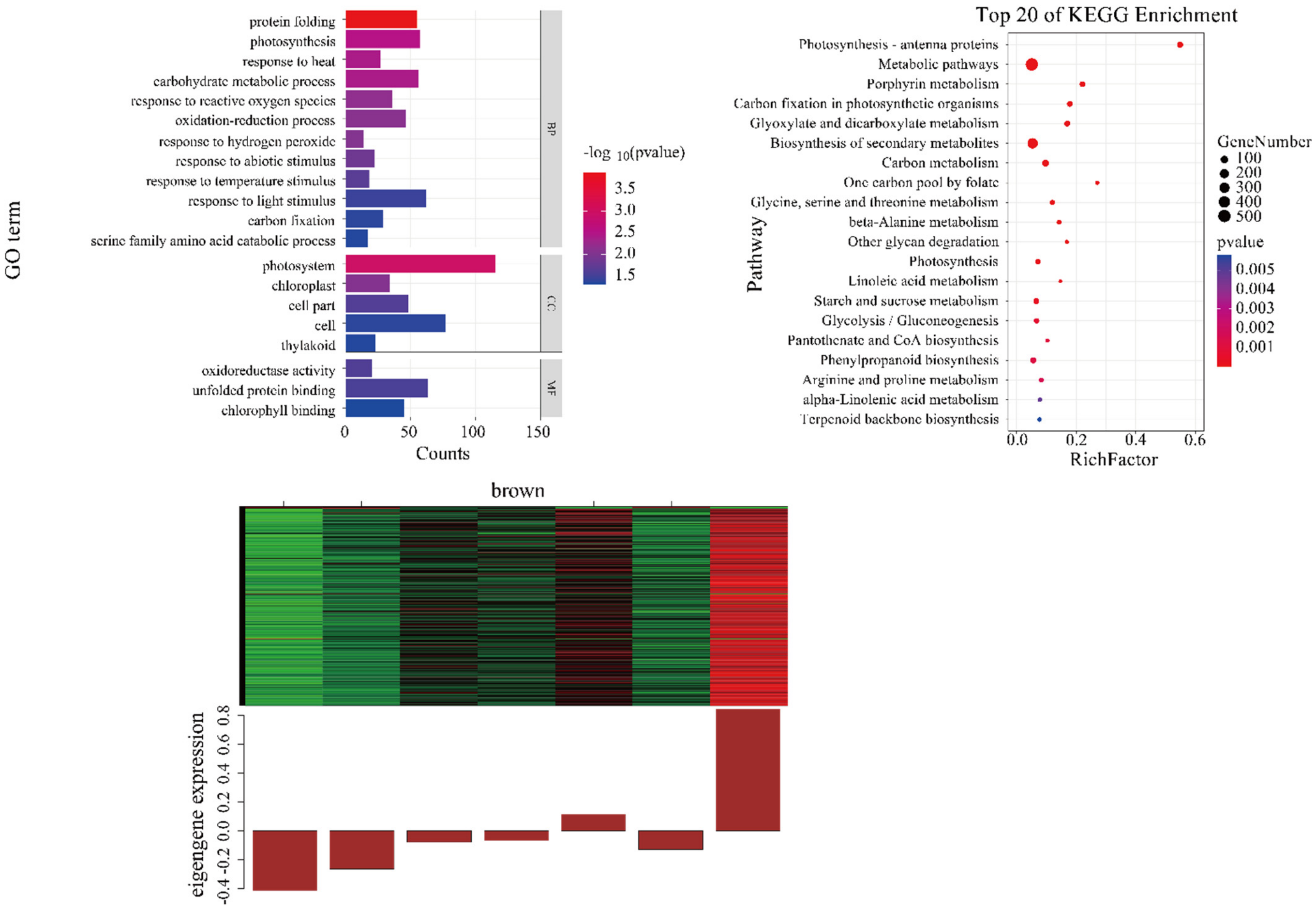

Supplementary Figure S4 MEbrown-related genes and their annotations. **A,** Significant GO terms in MEbrown. **B,** Significant KEGG terms in MEbrown. **C**, Eigengene expression during each treatment period in MEbrown; heatmap from green to red for expression from high to low, respectively.

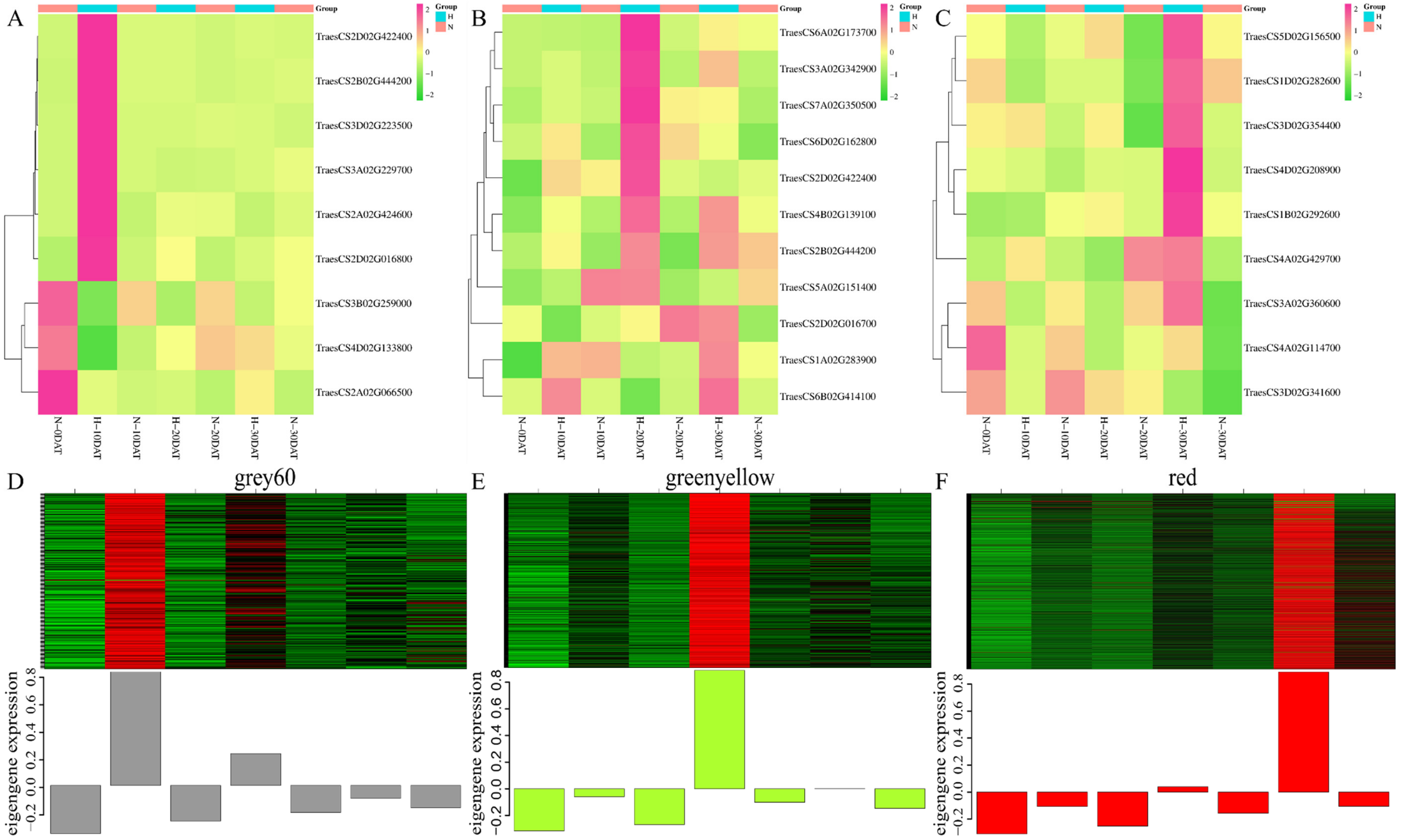

Supplementary Figure S5 Heat map displaying the expression of genes related to lignin synthesis in the MEgrey60 (A), MEgreenyellow (B), and MEred modules (C). The color gradient indicates the varying levels of gene expression. Eigengene expression during each treatment period in MEgrey60 (D), MEgreenyellow (E), and MEred modules (F); heatmap from green to red for expression from high to low, respectively.
