# Supplementary material for: Deciphering High-Temperature-Induced Lignin Biosynthesis in Wheat through Comprehensive Transcriptome Analysis
Source: Plants (Basel). 2024 Jul 3;13(13):1832. doi: 10.3390/plants13131832 (PMC11243994; doi:10.3390/plants13131832)
Supplement: Supplementary file 1 [file plants-13-01832-s001.zip › Supplementary materials File S1 Transcriptome analysis.pdf]

Methods S-1

Transcriptome analyses

RNA extraction and library preparation

Total RNA was extracted using the TRIzol Reagent (Vazyme, USA). RNA quality was checked by RNase-free agarose gel electrophoresis and then verified using an Agilent 2100 Bio-analyzer (Agilent Technologies, Santa Clara, CA, United States). mRNA was enriched with magnetic beads with Oligo (dT). Fragmentation buffer was then added to cleave the mRNA into small fragments. The first-strand cDNA was synthesized with random hexamers-primed, then the second-strand cDNA was synthesized using dNTPs and DNA polymerase I, and the double-strand cDNA was purified using AMPure XP beads. The purified double-stranded cDNA was then subjected to end repair, A-tailed, and ligated with sequencing adapters, followed by fragment size selection with AMPure XP beads, and finally, PCR enrichment to obtain the final cDNA library. Following agarose gel electrophoresis and extraction of cDNA from gels, the cDNA fragments were purified and enriched by PCR to construct the final RNA-Seq libraries, which were sequenced on the Illumina HiSeq X Ten platform using the paired-end mode. Three biological replicates were performed for each sample.

Transcriptome analysis

Raw RNA-Seq reads were filtered to remove low-quality sequences (Q20<85%), reads with more than 5% N bases (bases unknown), and reads containing adaptor sequences, using an in-house Perl script. The cleaned reads were mapped to the wheat reference genome of Chinese Spring (http://plants.ensembl.org/Triticum_aestivum/Info/Index) using HISAT2. Uniquely mapped reads were extracted, and gene expression was quantified by featureCounts and the number of fragments mapped to the exons of each gene. The expression of each gene in each sample was normalized to FPKM (expected number of Fragments Per Kilobase of transcript sequence per Millions of base pairs sequenced). At least two of the three biological replicates were considered to have gene expression detected in the samples (FPKM > 0). Differentially expressed genes were identified using DESeq2 and P-value corrected using false discovery rate (FDR) for multiple assays. In our study, genes with FDR<0.05 and fold change>2 were set as differentially expressed genes (DEGs). GO terms and KEGG pathways with corrected P-value < 0.05 were considered significantly enriched in the differential expressed genes.

Bioinformatic analysis

Cluster analysis

Cluster 3.0 (http://bonsai.hgc.jp/~mdehoon/software/cluster/software.htm) and Java Treeview software (http://jtreeview.sourceforge.net) were used to perform hierarchical clustering analysis. Euclidean distance algorithm for similarity measure and average linkage clustering algorithm (clustering uses the centroids of the observations) for clustering were selected when performing hierarchical clustering. A heat map was often presented as a visual aid in addition to the dendrogram.

GO annotation

The protein sequences of the selected differentially expressed proteins were locally searched using the NCBI BLAST+ client software (NCBI-blast-2.2.28) and InterProScan to find homologous sequences, then gene ontology (GO) terms were mapped and sequences were annotated using the software program Blast2GO. The GO annotation results were plotted by R scripts.

KEGG annotation

Following annotation steps, the studied proteins were blasted against the online Kyoto

Encyclopedia of Genes and Genomes (KEGG) database (http://geneontology.org/) to retrieve their KEGG orthology identifications and were subsequently mapped to pathways in KEGG.

Enrichment analysis

Enrichment analyses were applied based on Fisher' exact test, considering the whole quantified proteins as a background dataset. Benjamini- Hochberg correction for multiple testing was further applied to adjust derived p-values. And only functional categories and pathways with p-values under a threshold of 0.05 were considered significant.
